# Late ribosomal protein localization in *Arabidopsis thaliana* differs to that in *Saccharomyces cerevisiae*

**DOI:** 10.1002/2211-5463.12487

**Published:** 2018-07-25

**Authors:** Denise Palm, Deniz Streit, Maike Ruprecht, Stefan Simm, Christian Scharf, Enrico Schleiff

**Affiliations:** ^1^ Institute for Molecular Biosciences Goethe University Frankfurt am Main Germany; ^2^ Buchman Institute for Molecular Life Sciences Goethe University Frankfurt am Main Germany; ^3^ Frankfurt Institute of Advanced Studies Frankfurt am Main Germany; ^4^ Department of Otorhinolaryngology, Head and Neck Surgery University of Greifswald Germany

**Keywords:** Arabidopsis thaliana, eukaryotic system, intracellular protein localization, ribosomal proteins, ribosome biogenesis

## Abstract

Ribosome biogenesis is essential for cellular function and involves rRNA synthesis, rRNA processing and modification, and ribosomal protein assembly. Ribosome biogenesis factors and small nucleolar RNA assist these events. Ribosomal maturation takes place in the nucleolus, the nucleoplasm, and the cytosol in a coordinated and controlled manner. For example, some ribosomal proteins are thought to be assembled in the cytoplasm based on the observations in *Saccharomyces cerevisiae*. Here, we used cellular fractionation to demonstrate that cleavage of the 20S intermediate, the precursor to mature 18S rRNA, does not occur in the nucleoplasm of *Arabidopsis thaliana*. It most likely occurs in the cytoplasm. Further, we verified the proposed localization of RPS10e, RPS26e, and RPL24a/b in the nucleus and RPP1 in the nucleolus of *A. thaliana* by ribosome profiling, immunofluorescence, and analysis of the localization of GFP fusion proteins. Our results suggest that the order of events during ribosomal protein assembly in the ribosome biogenesis pathway differs between plants and yeast.

**Abbreviations**
(RP)L: ribosomal protein of the large subunit
(RP)P: ribosomal protein of the large subunit
(RP)S: ribosomal protein of the small subunit
RBF: ribosome biogenesis factor
RP: ribosomal protein
snoRNA: small nucleolar RNA

Transcription of 35S rRNA (yeast) or 45/47S (mammals) precursor is catalyzed by RNA polymerase I 1, 2, 3, 4, while 5S rRNA is transcribed independently by RNA polymerase III 5. The transcription of 35S rRNA in the nucleolus leads to the formation of the initial preribosomal complex, known as the 90S preribosomal particle 4. The maturation of 35S pre‐rRNA to 18S, 5.8S, and 25S rRNA; the rRNA modifications; and the assembly of ribosomal proteins (RP) are regulated by different ribosome biogenesis factors (RBFs) and small nucleolar RNA (snoRNA) 4, 6.

Ribosome biogenesis culminates in the cytoplasm by the maturation of two ribosomal subunits followed by an ultimate quality control process. In yeast, the cytoplasmic events of 40S ribosomal subunit maturation include the assembly of RPS10e (herein S10e) and formation of the 80S‐like complex for the quality control events 7. The latter induces cleavage of the pre‐18S‐rRNA at site D (D‐cleavage) by the endonuclease Nob1 8. The assembly of S26e is discussed as last step of the maturation of the small ribosomal subunit 7. During 60S maturation, the RPs L10 9, L24a/b 10, L29 11, L40 12, P0, P1, and P2 13 are incorporated into the ribosomal subunit in the cytoplasm. The assembly of L10 and L40 occurs last and requires the dissociation of Nmd3 by the function of Lsg1 9. Thus, while in fungi, at least 12 RPs (S1, S4, S6, S7, S8, S9, S11, S13, S17, S28, L4, and L13) are assembled in the 90S preribosomal particle 4, 14, nine RPs are thought to be associated with the cytoplasm as part of the final maturation and the quality control cycle.

However, ribosome assembly in plants 15, 16, 17, 18 or mammals 19, 20 was found to be in parts distinct from that in yeast. For example, Lsg1 involved in the release of Nmd3 in the cytosol of yeast is localized in the nucleoplasm of plants 21. Even more remarkable, a recent study of the protein content of the nucleolus and the nucleus by fractionation and proteome analysis revealed a putative localization of all ribosomal proteins in the nucleus 22. This observation suggests that a different quality control mode operates in plants.

To explore these differences between fungi and plants, we selected four ribosomal proteins from *Arabidopsis thaliana,* namely S10e, S26e, L24a/b, and P1, the counterparts of which in yeast are known to be assembled in the cytoplasm 17, 22, 23, and determined their subcellular localization by fluorescence microscopy. We confirmed the localization of S10e, S26e, and L24a in the nucleoplasm and of P1 in the nucleolus. The consequence for the regulation of ribosome assembly in plants is discussed.

## Material and methods

### RNA isolation and northern blot analysis

The cellular fractionation of cells was performed as described 21, 22. For this purpose, *A. thaliana* root cell suspension cultures were grown for 5 days in the dark at 24 °C while shaking at 150 r.p.m. After separation of cytoplasmic and nuclear fractions, RNA was isolated and analyzed by northern hybridization using previously described probes 21, 24, 25. The signal density was quantified with ImageJ (imagej.net).

### Cloning of GFP constructs

The coding sequences of RPS10e (At5g41520), RPS26e (At3g56340), RPL24a (At2g36620), and RPL24b (At3g53020) and the three co‐orthologous genes of RPP1 (At1g01100, At4g00810, and At5g47700) 23 were amplified by conventional PCR with *A. thaliana* cDNA template 25 using the oligonucleotides listed in Table 1. The amplified DNA was restricted by KpnI and SpeI and cloned in pRTds‐GFP vector 21, 24 to generate N‐ and C‐terminal GFP fusion constructs.

**Table 1:** Oligonucleotides

RP	AGI	Oligo	Sequence
L24a	At2g36620	L24A_F_KpnI	ATATTAGGTACCATGGTTCTCAAGACTGAGCTTTGCCG
		L24A_R_SpeI	ATTTACTAGTACGTCTGCCTCCACCACCACCC
L24b	At3g53020	L24B_F_KpnI	ATATTAGGTACCATGGTTCTCAAGACGGAGCTTTGTCG
		L24B_R_SpeI	ATTTACTAGTGCGTTTGCCACCACCACCTCCCACC
P1.1	At1g01100	P1.1_F_KpnI	ATATTAGGTACCATGTCGACAGTTGGAGAGCTTGC
		P1.1_R_SpeI	ATTTACTAGTGTCAAACAAACCGAAACCC
P1.2	At4g00810	P1.2_F_KpnI	ATATTAGGTACCATGTCGACAGTCGGAGAACTTGCTTGC
		P1.2_R_SpeI	ATTTACTAGTATCGAACAAGCCGAAACCAAGATCTCC
P1.3	At5g47700	P1.3_F_KpnI	ATATTAGGTACCATGTCGACAGTGGGAGAGCTCGC
		P1.3_R_SpeI	ATTTACTAGTATCGAACAAGCCAAAACCCAAATCTCC
S10e	At5g41520	S10_F_KpnI	ATATTAGGTACCATGATCATATCAGAGACTAACCGCCG
		S10_R_SpeI	ATTTACTAGTAGGAAGATCAGATCCAGCAGCACCACC
S26e	At3g56340	S26_F_KpnI	ATATTAGGTACCATGACTTTCAAGCGCAGGAATGG
		S10_R_SpeI	ATTTACTAGTGGCACGAGGAGCAGCAGGAGCACC

### Protoplast isolation and transformation

Protoplast isolation from *A. thaliana* and their transformation were previously described 21, 24, 26. We cotransfected pRTdS‐Fib2‐mCherry for expression of a nucleolar marker 24. Protoplasts were incubated overnight (12 h, 25 °C, continuous light) 21, 26 before further processing.

### Analysis of protein distribution by confocal laser‐scanning microscopy

Transformed protoplasts were spotted on an object slide and analyzed using a Leica SP5 confocal microscope 26. GFP and mCherry were excited at 488 and 568 nm, respectively. GFP and mCherry fluorescence were detected at 505–525 nm and 580–610 nm, respectively. Chlorophyll autofluorescence was excited at 488 nm and fluorescence monitored at 660–710 nm.

### Analysis of protein distribution by immunofluorescence

Indirect immunofluorescence of *A. thaliana* mesophyll protoplasts and isolated nuclei were performed as described 27. Cells and isolated nuclei were incubated overnight with antibodies against S10e (αS10e) diluted 1 : 5000 in PBS‐1% bovine serum albumin. After washing, cells and nuclei were incubated with the secondary fluorochrome‐labeled antiserum in a 1 : 1000 dilution for 1.5 h in the dark. As a control, protoplasts and isolated nuclei were stained with 4′,6‐diamidino‐2‐phenylindole (DAPI) for localization of nuclei.

### Ribosome profiling

For the analysis of GFP‐fused ribosomal protein association with ribosomal subunits, GFP fusion proteins were expressed for 16 h in the dark in protoplasts of *A. thaliana* cell suspension culture. Subsequently, protoplasts were lysed and loaded onto a continuous 10–50% (w/v) sucrose gradient and centrifuged for 18 h at 100 000 ***g*** in a TST41.14 rotor. Collection of fractions was performed as described 25.

## Results and Discussion

### D‐cleavage in plants occurs in the cytoplasm

The final steps of ribosome biogenesis in fungi occur in the cytoplasm 28. Major hallmarks of 40S maturation are cleavage of the 20S rRNA at site D yielding the 18S rRNA and assembly of S10e and S26e. Further, 60S maturation in the cytoplasm involves displacement of several RBFs and assembly of seven ribosomal proteins. The last steps of 60S maturation are the release of Nmd3 catalyzed by Lsg1 and the assembly of L40 and L10. Contrary to this, the orthologue of Lsg1 in *A. thaliana* involved in maturation of the large subunit, namely Lsg1‐2, is targeted to the nucleus 21. This might indicate that the mechanism of Nmd3 regulation in plants is different to that in yeast.

**Figure 1:**
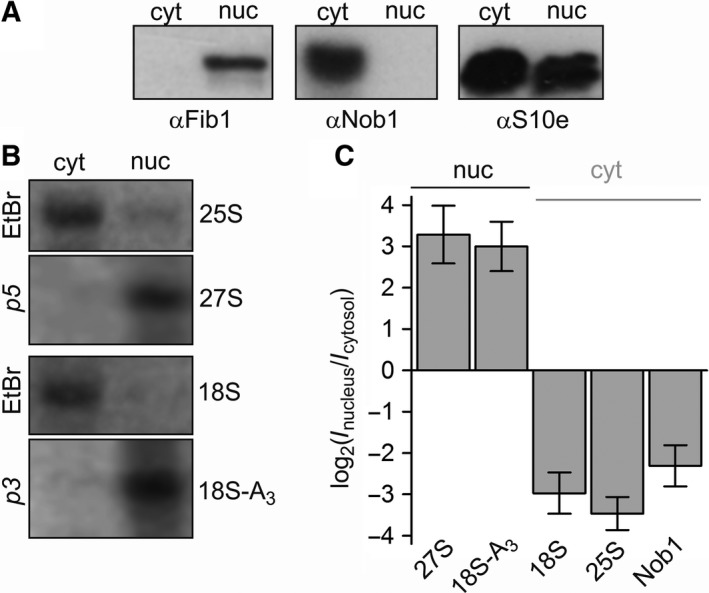
Localization of the D‐cleavage of the 18S rRNA. (A) Cytosolic (cyt) and nuclear fractions (nuc) of *A. thaliana* were subjected to SDS/PAGE followed by western blotting and immunodecoration with indicated antibodies. (B) Same fractions as in (A) were subjected to agarose gel. 27S and 18S‐A_3_ were detected by northern blotting with radioactive probes (p5 and p3, respectively 21), and 25S and 18S were visualized by ethidium bromide staining. (C) The density of the signal was quantified and the ratio of the signal (I) in the nuclear and the cytosolic fraction is expressed as logarithm of two for better representation. Error bars indicate standard deviation of independent experiments (*n* > 5).

Consequently, we analyzed the distribution of 18S rRNA between cytosol and nucleus in *A. thaliana* (Fig. 1). The efficiency of fractionation was confirmed by immunodecoration with antibodies against fibrillarin serving as nucleolar marker and Nob1 as cytoplasmic marker (Fig. 1A, 24). We analyzed the distribution of 27S and 18S‐A_3_ transcripts by northern blot (Fig. 1B) with previously described probes 21. Quantification yielded an at least ten‐fold higher concentration of both rRNA precursors in the nuclear fraction than in the cytoplasmic fraction (Fig. 1C). Next, we analyzed the distribution of the mature 18S and 25S rRNA by agarose gel migration (Fig. 1B). Quantification yielded an about ten‐fold higher concentration of the two rRNA in the cytosolic fractions than in the nuclear fractions (Fig. 1C). This result demonstrated that final processing apparently does not occur in the nucleoplasm. This observation together with the cytosolic localization of Nob1 suggests that the final processing of 18S rRNA occurs neither in the nucleolus nor in the nucleoplasm, but most likely in the cytosol. This is similar to the cytosolic 18S rRNA maturation in yeast. However, the processing of 20S pre‐rRNA at site D is likely coordinated with the export of small subunit, because the 18S‐A_3_ rRNA precursor is barely if at all detectible in the cytoplasm.

### Ribosomal proteins are targeted to the nucleus of A. thaliana mesophyll cells

The nuclear proteome analysis of *A. thaliana*,* M. truncatula*, rice, barley, and tomato 22, 29, 30, 31, 32 led to identification of ribosomal subunit RPs that are usually of cytoplasmic in nature in yeast. In line with these observations, western blot analysis revealed that the S10e protein was detectable in nuclear fractions of *A. thaliana* (Fig. 1A). We selected four RPs and their orthologues to confirm the observed nuclear localization, namely two RPs of the small (S10e and S26e) and two of the large ribosomal subunit (L24a/b and P1) that are known in yeast to be assembled into ribosomes in the cytoplasm.

**Figure 2:**
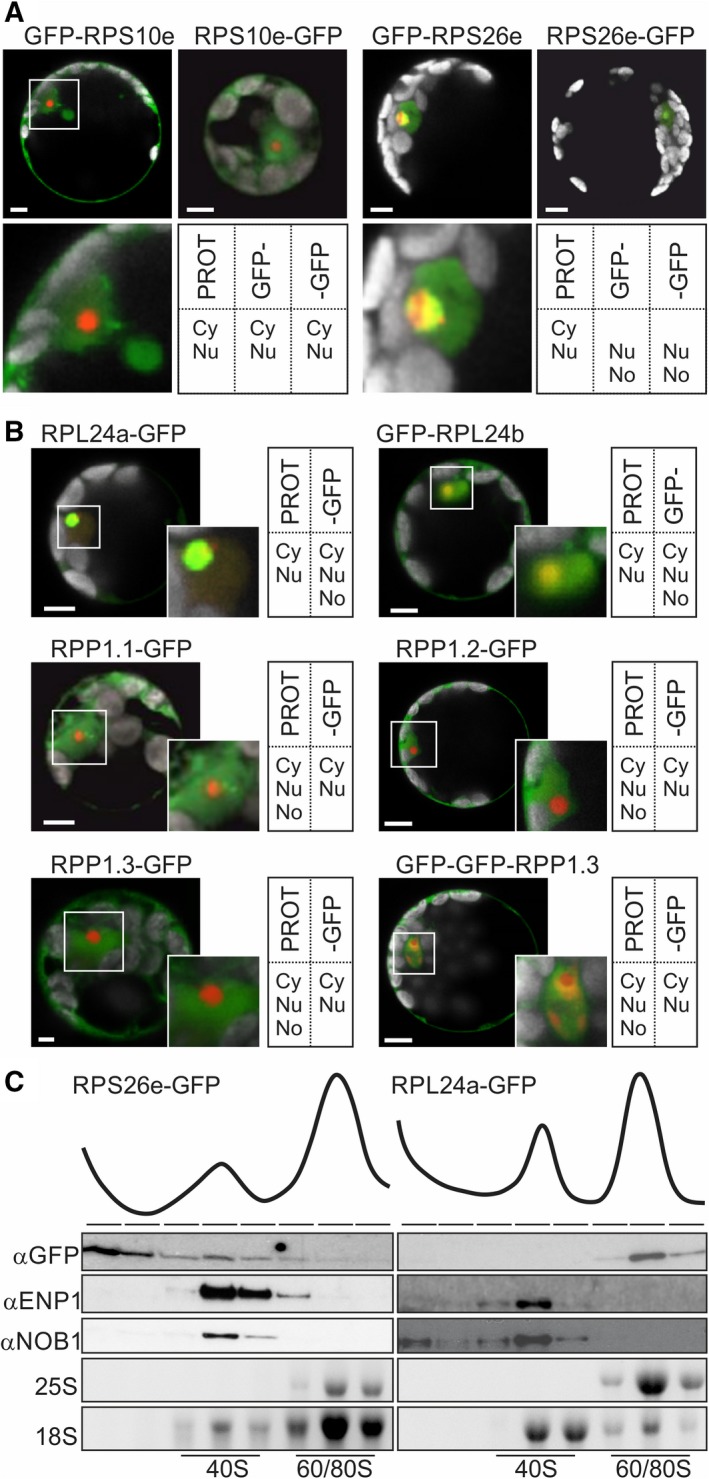
Localization of RPs in protoplasts. (A,B) GFP fusion constructs of S10e or S26e (A) or of L24a or P1 (B) were cotransformed with the nucleolar marker Fib2‐mCherry into mesophyll protoplasts from *Arabidopsis thaliana*. The overlay of GFP (green), Fib2‐mCherry (red), and chlorophyll autofluorescence signal (gray) is shown for a representative protoplast. The scale bar is 5 μm. PROT means localization of the protein identified by proteomic analysis, GFP‐ means the fusion protein with N‐terminal protein, and ‐GFP means the fusion protein with C‐terminal GFP protein. Cy indicates observed cytosolic localization, Nu indicates localization in the nucleus, and No indicates localization in the nucleolus. (C) Protoplasts were transformed with RPS26e‐GFP (left) or RPL24a‐GFP. After expression, cells were solubilized and fractionated by sucrose density gradient centrifugation. The absorption profile is shown on top. The indicated fractions were subjected to western blot analysis using GFP (top panel), ENP1 (second panel), or NOB1 (third panel) antibodies. The rRNA content of the same fractions was determined by agarose gel separation and ethidium bromide staining. 25S and 18S rRNA are shown in panels four and five.

The coding sequence of S10e was fused to the 3′ or 5′ end of the coding region of GFP (GFP‐RPS10e or RSP10e‐GFP, respectively). Both proteins were expressed in mesophyll protoplasts isolated from *A. thaliana* leaves. We cotransformed these cells with a plasmid coding for atFIB2‐mCherry 21 to visualize nucleolus. We detected a nuclear and cytosolic localization by confocal fluorescence imaging, irrespective of the construct used (Fig. 2A). This is in line with the protein distribution observed in proteomic studies 22.

Inspecting the localization of GFP‐RPS26e or RPS26e‐GFP by the approach described for S10e, we observed a nucleolar and a nuclear localization, while fluorescence in the cytoplasm was not observed. Nevertheless, our result is in line with proteome analysis of the nucleus isolated from *A. thaliana*, rice, and tomato (Table 2).

**Table 2:** Identification of RPS in exemplary proteomic studies of the nucleus of indicated plants

RP	*A. thaliana* 22	*M. truncatula* 29	Rice 30	Barley 31	Tomato 32
S10e	+		+	+	+
S26e	+		+		+
L10	+	+	+	+	+
L24	+		+	+	+
L29	+				
L40				+	
P0	+	+	+		+
P1	+		+		+
P2	+		+		

Next, we used C‐ and N‐terminal GFP fusion constructs of RPL24a and RPL24b for protoplast transformation, respectively. Remarkably, GFP fluorescence was detected in the nucleolus, nucleus, and cytoplasm (Fig. 2B). This shows that nuclear targeting was not dependent on the position of GFP. Again, this observation confirms the nuclear localization of the proteins determined by proteomics in *A. thaliana* and other plant species (Table 2). The nucleolar GFP fluorescence observed for both proteins is likely specific, because RPS10e fused to GFP did not yield nucleolar GFP fluorescence (Fig. 2A).

Further, we analyzed the localization of the three orthologues of P1. Proteomic studies of *A. thaliana*, rice, and tomato identified this protein in the nucleus (Table 2) and even a nucleolar localization was suggested 22. Using GFP fusion proteins, we observed GFP fluorescence in the nucleoplasm and cytoplasm (Fig. 2B). On the example of the orthologue P1.3, we demonstrated that nuclear localization is not dependent on the position of the GFP fusion. Moreover, the transport appears to be active as a double GFP fusion exceeding the size limit of the nuclear translocation pore 33 confirming nuclear localization as well (Fig. 2B).

To confirm the association of S26e and L24a with ribosomal complexes, protoplasts were transformed with RPS26e‐GFP or RPL24a‐GFP. After expression, cells were solubilized and ribosome profiling was performed based on sucrose density centrifugation. We observed a fraction of RPS26e‐GFP bound to 40S ribosomes, while a portion was observed in the soluble fraction as well (Fig. 2C, left). The latter observation and the absence of a strong GFP signal in the cytoplasm after expression of RPS26e‐GFP might suggest that GFP‐tagged S26e can be assembled into pre‐40S ribosomes, but the GFP tag interferes with the assembly of functional ribosomes in the cytoplasm. Nonfunctional ribosomal units are degraded explaining the low abundance of GFP fluorescence 34. In turn, RPL24a‐GFP was largely assembled into ribosomes (Fig. 2C, right).

**Figure 3:**
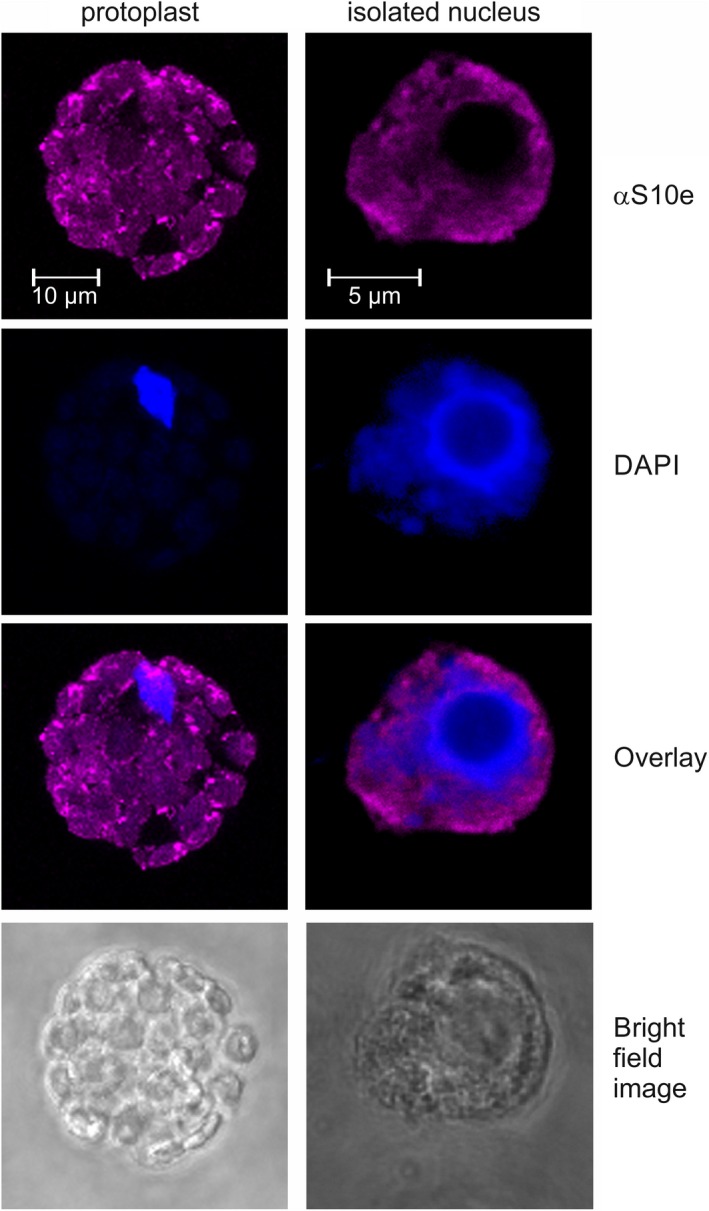
Localization of RPS10e by immunofluorescence. Protoplasts (left) or isolated nuclei (right) were incubated with antibodies against S10e (αS10e) and DAPI. The immunofluorescence (top), the DAPI staining (second panel), the overlay of both signals (third panel), and the bright field image (bottom) are shown for a representative sample. Scale bars are indicated.

Next, we used antibodies against S10e to detect the endogenous protein in cells by immunofluorescence. Staining entire cells yielded a strong signal in the cytoplasm (Fig. 3, left), likely representing S10e in cytosolic ribosomes. When isolated nuclei were incubated with antibodies against S10e, we observed a signal in the nucleoplasm, but not in the nucleolus (Fig. 3, right). These results are consistent with the localization of S10e fused to GFP as well.

## Conclusion

In yeast, the final maturation of the ribosomal subunits including the assembly of several RPs occurs in the cytoplasm 6, 7, 8, 9, 10, 11, 12, 13, 14, 28. Our findings (Figs 1, 2, 3) and recent proteome analyses of nuclear fractions 22 suggest that RPs involved in late events of plant ribosome biogenesis differ from yeast in terms of localization 28. Our conclusion is based on previous analyses of nuclear proteome of different plants 22, 29, 30, 31, 32, the localization of GFP fusion proteins (Fig. 2A,B) that are actively targeted as confirmed by the double GFP‐tagged RPP1 (Fig. 2A), association with ribosomal subunits as shown for S26e and L24a (Fig. 2C), and by the localization of endogenous S10e (Fig. 3).

**Figure 4:**
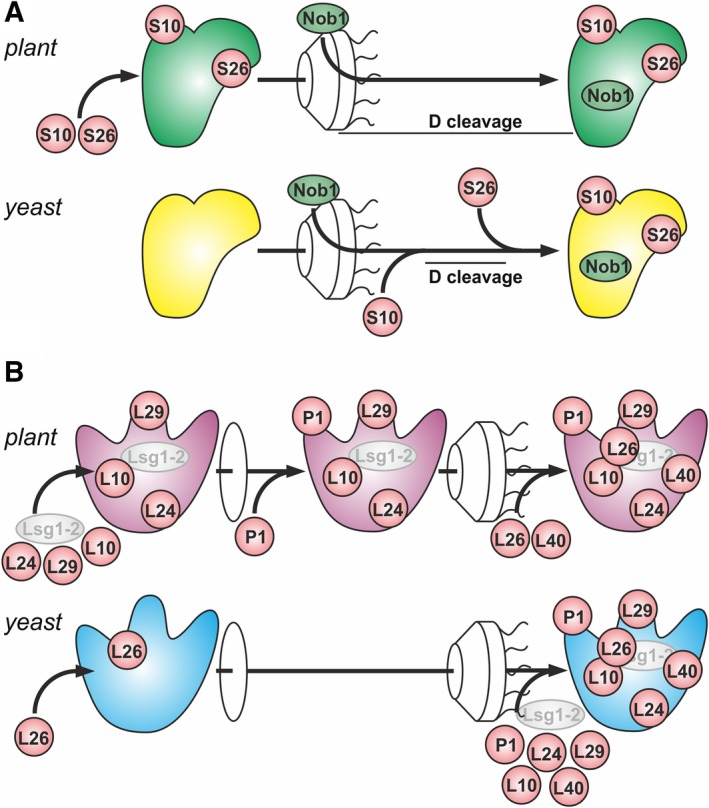
Timing of the RP association with preribosomal complexes in plants. Shown are the events of small (A) or large (B) ribosomal subunit maturation for plants (top) and yeast (bottom) that are assigned to occur in the cytosol in yeast. The steps in the nucleolus (before disk), in the nucleoplasm (between disk and nuclear pore model), and in the cytoplasm (after nuclear pore model) are indicated. Positioning of the proteins is just to illustrate the order of events and not according to ribosomal structures.

With respect to 40S maturation in plants (Fig. 4A), we detected S10e in the nucleus (Figs 2, 3; Table 2). Yeast S10 is assembled into ribosomes in the cytoplasm 7. It is likely that the assembly occurs in the nuclear pore as yeast S10 is important for the export of the small subunit from the nucleus 14. Similarly, *Arabidopsis* S26e is already present in the nucleoplasm (Fig. 2, Table 2), while in yeast, it is assembled into ribosomes of cytoplasm markedly after release of Pno1 7, 35 and D‐cleavage. 14. In line with a nuclear assembly of S26e into ribosomes in plants, mass spectrometry revealed that Pno1 was solely detected in nucleolar and nuclear fractions of *A. thaliana* 22.

Mature 18S rRNA is exclusively present in the cytoplasm of *A. thaliana*, while the 18S‐A_3_ transcript is observed in the nucleoplasm (Fig. 1). This suggests that pre‐rRNA cleavage at site D likely is associated with the transport from nucleoplasm to cytoplasm. This would resemble the path in yeast 8, 14. However, it remains to be established whether D‐cleavage occurs in the vicinity of the nuclear pore complex.

Maturation of the 60S ribosomal subunit differs between plants and yeast as well (Fig. 4B). Yeast L26 is assembled into ribosomes in the nucleus 36, while the plant protein was only detected in the cytoplasm 22. In turn, many ribosomal proteins assembled into ribosomes in the cytoplasm in yeast were identified in the nucleus of various plant species (Table 2, 22, 29, 30, 31, 32). At least L24, L29, and L10, as well as Lsg1‐2, are targeted to the nucleolus (Fig. 2, 21, 22). P1 associates with the precursor of the 60S subunit in the nucleoplasm (Fig. 2). In contrast, yeast L10, L24, L29, and P1 are incorporated into ribosomes in the cytoplasm 9, 10, 11, 13. For example, L24 replaces RLP24 during yeast ribosomal maturation 10, while alternative pattern of L24 incorporation into ribosomes is evident as RLP24 is only found in the nucleolus of *A. thaliana* 22. However, L40 is an example where association with the ribosome is similar between yeast and *A. thaliana* (Table 2; 12).

Thus, we provide experimental evidence for differences in the order of events during final maturation of ribosomes in plants when compared to yeast. Whether the initial association of the ribosomal proteins with the rRNA occurs at the site of function in the mature ribosome remains to be established. Moreover, the existing results do not contradict the formation of 80S‐like particle for quality control in the cytoplasm 7 although its formation remains to be shown for plant systems.

## Author contributions

ES and CS conceived the project. DP, DS, and ES designed the experiments, which were performed by DP, DS, and MR. ES and SS carried out the theoretical analysis. DP, DS, and ES wrote the manuscript, and all authors approved the manuscript.
